# Predictive Value of Musculoskeletal Fitness for Cardiovascular Risk Factors in Adolescents with Congenital Heart Disease: A Cross-Sectional Study

**DOI:** 10.3390/jcm15082863

**Published:** 2026-04-09

**Authors:** Kunyu Hao, Craig A. Williams, Alan R. Barker, Curtis A. Wadey, Jan Müller, Renate Oberhoffer, Laura Willinger

**Affiliations:** 1 The Children’s Health and Exercise Research Centre, Public Health and Sport Sciences, Faculty of Health and Life Sciences, St. Luke’s Campus, University of Exeter, Heavitree Road, Exeter EX1 2LU, UK; kh704@exeter.ac.uk (K.H.); a.r.barker@exeter.ac.uk (A.R.B.); 2 Research and Improvement, Hampshire and Isle of Wight Healthcare NHS Foundation Trust, Moorgreen Hospital, Southampton SO30 3JB, UK; curtis.wadey@southernhealth.nhs.uk; 3 School of Medicine and Health, Technical University Munich, Am Olympiacampus 11, 80809 München, Germany; j.mueller@tum.de (J.M.); renate.oberhoffer@tum.de (R.O.); laura.willinger@tum.de (L.W.)

**Keywords:** congenital heart disease, adolescents, musculoskeletal fitness, cardiovascular risk factors

## Abstract

**Objectives:** We aimed to investigate the association between musculoskeletal fitness (MF) and cardiovascular risk factors in adolescents with congenital heart disease (ConHD). **Methods:** This cross-sectional study included 355 adolescents with ConHD (median age 12.4 years [range: 5.7–21.7]; 43.4% female). Participants completed musculoskeletal fitness (MF) tests, including handgrip strength (HGS), curl-ups, push-ups, and trunk lifts, and underwent an assessment of anthropometric indices, blood pressure, pulse wave velocity (PWV), and carotid intima–media thickness (cIMT). To account for body size, HGS was allometrically scaled to body mass with adjustment for age, sex, and ConHD severity. Clustered MF was derived by calculating z-scores for allometric HGS, curl-ups, push-ups, and trunk lifts. **Results:** Allometric HGS was inversely associated with anthropometric indices: waist circumference [WC] (β = −4.467, *p* = 0.014), waist-to-hip ratio [WHR] (β =−0.039, *p* = 0.005), waist-to-height ratio [WHtR] (β = −0.052, *p* = 0.001), and BMI (β = −3.115, *p* = 0.001). Push-ups were inversely related to all anthropometric indices (*p* < 0.05). Trunk lift showed positive associations with multiple anthropometric indices except WHR (all *p* < 0.05). Clustered MF was negatively associated with WHR (β = −0.004, *p* = 0.008) and WHtR (β = −0.006, *p* = 0.001). HGS (β = 0.18, *p* = 0.033), push-ups (β = 0.004, *p* = 0.041), and clustered fitness (β = 0.028, *p* = 0.006) were inversely associated with PWV. **Conclusions:** Systematically increasing MF in rehabilitation may provide a feasible strategy to mitigate CVD risk in adolescents with ConHD.

## 1. Introduction

Due to abnormalities in cardiac and/or intrathoracic vessel development [1], only 32% of adolescents with congenital heart disease (ConHD) achieve ideal cardiovascular health, and they continue to face potential challenges in managing long-term cardiovascular disease (CVD) risks [2,3]. Current physical activity guidelines recommend that adolescents with ConHD should aim to approach the standards outlined for the general population, an average of 60 min of moderate-to-vigorous physical activity (MVPA) per day, to manage CVD risk [4]. Physical activity guidelines also recommend engaging in activities that promote muscular fitness [4]. Musculoskeletal fitness (MF) reflects the neuromuscular system’s overall capacity to generate and coordinate strength and movement, which has been associated with multiple health benefits [5]. However, few studies have examined the association between MF and the risk of CVD in adolescents with ConHD.

Studies examining the association between MF and CVD risk have focused primarily on healthy adolescents. Low MF in adolescents was found to be independently associated with higher CVD risk [6]. Evidence from a recent systematic review in healthy adolescents demonstrated that higher MF was consistently associated with a lower waist circumference (WC) [12/15 studies], whereas findings regarding its relationship with blood pressure (BP) were inconsistent. Together, these patterns indicate that MF may relate more to anthropometric than hemodynamic CVD risk factors, at least in healthy adolescents. For adolescents with ConHD, this association is particularly important in preventing cardiovascular disease, as they already exhibit decreased metabolic efficiency and reduced vascular elasticity in childhood [7,8].

Two recent studies on CVD risks in adolescents with ConHD primarily emphasised the role of MVPA [9,10]. Higher levels of MVPA were associated with lower aortic pulse wave velocity (PWV) and had no significant association with central BP [9,10]. PWV and central BP are also strong predictors of CVD risks [11]. Given the bidirectional relationship between MVPA and MF, it is plausible that MF may also relate to PWV [12]. Exploring these associations may support the development of more comprehensive and personalised PA in adolescents with ConHD. However, existing research has predominantly focused on physical activity (e.g., MVPA), with comparatively limited attention given to MF, particularly regarding its potential role as a predictive indicator of CVD risk in adolescents with ConHD. This study aims to investigate the association between MF and key CVD risk factors (WC, hip circumference [HC], waist-to-hip ratio [WHR], waist-to-height ratio [WHtR], body mass index [BMI], peripheral systolic blood pressure [SBP], peripheral diastolic blood pressure [DBP], central SBP and central DBP, PWV, heart rate [HR], and carotid intima media thickness [cIMT]) among adolescents with ConHD.

## 2. Materials and Methods

### 2.1. Study Design and Sample

This study employed a cross-sectional observational design and was conducted and reported in accordance with the STROBE (Strengthening the Reporting of Observational Studies in Epidemiology) guidelines for cross-sectional studies, ensuring methodological transparency and completeness [13,14]. The completed STROBE checklist is provided as Table S1.

This paper is based on data from the single-centre FOOTLOOSE (Functional outcomes in children and adolescents with congenital heart disease) study, which received ethics approval from the Medical Ethics Committee of the Technical University of Munich (project number 314/14 and date of approval 27 August 2014). At the commencement of this study in 2014 and according to the guidelines of the International Committee of Medical Journal Editors (ICMJE), this study was not required to be registered because it was not defined as a clinical intervention. However, subsequently in 2019, to enhance the visibility of the project and follow good practice for reporting studies, FOOTLOOSE was retrospectively registered: https://drks.de/search/en/trial/DRKS00018853/details—accessed on 30 September 2019.

Three hundred and fifty-five adolescents (201 males, 154 females) aged 12.4 years (Interquartile Range: 9.3–15.4) were included in the current study from the original study sample. Adolescents participating in the study and their parents/guardians signed a consent form. In this study, adolescents were included based on having the following data: (1) MF (handgrip strength [HGS], curl-ups, push-ups and trunk lifts); (2) BP (peripheral SBP, peripheral DBP, central SBP and central DBP); (3) PWV, HR, and cIMT; and (4) anthropometrics (WC, WHR, WHtR and BMI).

### 2.2. Musculoskeletal Fitness

HGS was measured using an analogue handgrip dynamometer (SH5001, Saehan, Changwon, Republic of Korea) in kilogrammes (kg). Each participant started with the right hand and executed three maximum isometric contractions of the forearm muscles, each lasting up to two seconds, interspersed with brief rest periods between trials. The same technique was subsequently executed with the left hand. Measurements were conducted in an upright standing posture, with the tested arm aligned parallel to the torso, shoulder slightly adducted, elbow flexed at 90°, forearm in a neutral position, and wrist positioned between 0–30° of extension and 0–15° of ulnar deviation [15]. Mean handgrip strength was calculated and analysed as the average of six trials. The mean value was used to improve measurement reliability and reduce variability across repeated trials.

To account for the influence of body mass, HGS was allometrically scaled for body mass using the equation below. The natural log-transformed values of HGS and body mass were calculated, and a log-linear regression was conducted to derive allometric exponent b, with adjustment for age, sex, and ConHD disease severity. *b* was calculated from the slope of the regression of log-transformed handgrip strength against log-transformed body mass within the study sample.

(1) $$Allometric\;scaled\;handgrip\;strength=\frac{handgrip\;strength\;(kg)}{{body\;mass\;(kg)}^{b}}$$

Participants completed curl-ups, push-ups, and trunk lifts to further assess upper body strength and core strength. During the push-up test, participants maintained a straight body position and flexed the elbows to at least 90°, continuing repetitions until volitional exhaustion or failure to maintain proper form. The total number of correctly completed repetitions was recorded. For the curl-up test, participants adopted a supine position with knees flexed (at 140°), extending their arms toward their knees. Each time the hands reached the knees was counted as one valid repetition, and the maximal number was recorded. For the trunk lift, participants lifted their upper body off the ground, and the vertical distance from the chin to the floor was measured. Two trials were performed, and the maximal value was used for analysis. The clustered MF score was calculated by summing the individual standardised z-scores for allometrically scaled HGS, curl-ups, push-ups, and trunk lift [6]. More detailed instructions are found in Table S2.

### 2.3. Anthropometrics

Height, body mass, WC and HC were measured following standardised procedures in accordance with the World Health Organisation [16]. WHR, WHtR and BMI were calculated: WHR was calculated as WC/HC, WHtR was calculated as WC/height, and BMI was calculated as body mass/height^2^ (kg·m^−2^). Weight status was classified using age- and sex-specific BMI-for-age z-scores according to the World Health Organisation growth reference [17]. Normal weight was defined as BMI-for-age between −2 and +1 SD, overweight as >+1 SD, and obesity as >+2 SD.

### 2.4. Cardiovascular Screening

PWV was analysed using an oscillometric measurement device (Mobil-o-Graph^®^, I.E.M Healthcare, Stolberg, Germany) [10]. Central BP was indirectly estimated with the ARCSolver Algorithm (Austrian Institute of Technology, Vienna, Austria) based on the recorded brachial pulse waves [18]. Measurements were performed at the left upper arm in a supine position after 5 min of rest, with the cuff size adjusted for individual arm circumference.

cIMT was assessed using B-mode ultrasound following standard guidelines [10]. Participants lay in a supine position with the head turned 45° away from the examined side. A semi-automated device (Cardio Health Station, Panasonic, Yokohama, Japan) was used to obtain four far-wall measurements of the common carotid artery at end-diastole, approximately 1 cm proximal to the bulb (left: 210°, 240°; right: 120°, 150°). The average of the four measurements was calculated and analysed.

### 2.5. Statistical Analyses

All data are expressed as the mean ± SD for normally distributed data, or median and IQR for non-normally distributed data. Normality was assessed using the Kolmogorov–Smirnov test, skewness/kurtosis values, and histogram inspection. Data are also stratified by ConHD severity (simple, moderate, and complex), defined according to the American College of Cardiology anatomical classification of lesion severity [19].

To evaluate the potential for systematic bias due to participant exclusion in the current study, we compared the differences between the final included participants and those not included in terms of sex, age, height, body mass, and MF by independent-samples *t*-tests and chi-square tests.

Regression models were used to examine the association between exposure variables, allometrically scaled HGS, curl-ups, push-ups, and clustered MF with outcomes, WC, HC, WHR, WHtR, BMI, peripheral SBP, peripheral DBP, central SBP, central DBP, HR, PWV, and cIMT. Age (years), sex (male/female), and ConHD severity (simple, moderate, and complex) were included as covariates. Separate models were run for each outcome after adjustment for age, sex, and ConHD severity. Regression output was reported using the standardised beta regression coefficient, 95% confidence intervals and the corresponding *p*-value. The normality and independence of residuals assumed by the regression model were visually checked, and multicollinearity was also examined.

To identify the potential dose–response of significant associations identified through the regression models, we used analysis of covariance (ANCOVA) to explore the relationship between the significant exposure variables highlighted in the regression model and CVD risk factors by quartile analysis after adjustment for age, sex, and ConHD severity. Significant effects were followed up using Bonferroni-adjusted pairwise comparisons.

All statistical analyses were conducted by IBM SPSS Statistics for Windows (SPSS 28.0; IBM Corporation, Armonk, NY, USA). Statistical significance was accepted as α of 0.05.

## 3. Results

**Figure 1:**
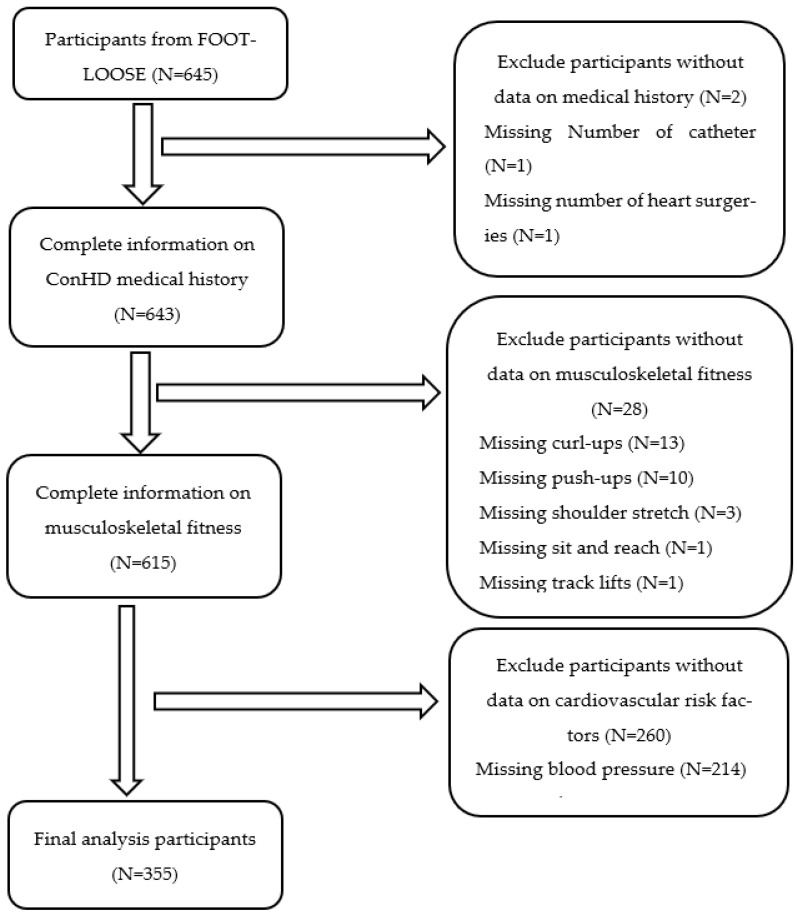
Flowchart of participant selection from FOOTLOOSE. FOOTLOOSE, functional outcomes in children and adolescents with congenital heart disease; ConHD, congenital heart disease.

Of the 645 participants included in the FOOTLOOSE cohort study, 355 participants were included in the final analysis (see Figure 1). A total of 290 participants were excluded from the study due to missing information on medical history (*n* = 2), incomplete MF data (*n* = 28), or incomplete measurements of CVD risk (*n* = 260). There were no significant differences between included and excluded participants for any variable except trunk lifts. Although the difference in trunk lifts reached statistical significance, the effect size was minimal (see Table S3).

The descriptive characteristics of the study sample are presented in Table 1. The cohort included 355 adolescents with ConHD, with a distribution across disease severity groups. The distribution of sex, age, cardiac diagnoses, medication use, and surgical history reflects the expected clinical heterogeneity of this population. Anthropometric, MF and CVD risk indicators demonstrated relatively broad variability across study samples. For example, handgrip strength ranged from 11.0 to 28.3 kg, curl-up performance from 4 to 28 repetitions, and BMI from 15.76 to 23.21 kg·m^−1^. These descriptive data provide the clinical and physiological context for the subsequent analyses.

### 3.1. Musculoskeletal Fitness and Cardiovascular Risk Factors

Results from the regression models for the association between MF and CVD risk factors are shown in Table 2. HGS and push-ups were significantly and inversely related to WC, WHR, WHtR, and BMI. A significant positive relationship was observed for HGS and PWV, as well as push-ups and PWV. No significant association between HGS and HC was observed. Curl-ups were significantly and inversely related to WHR, WHtR and BMI and not significantly related to HC, WC and PWV. There was no significant association between trunk lifts and WHR and PWV. Significant negative relationships were observed for clustered fitness with WHR and WHtR. Clustered fitness had a significant positive relationship with PWV. All MF exposure variables had no significant association with BP, HR and cIMT.

### 3.2. Musculoskeletal Fitness Quartiles and Cardiovascular Risk Factors

**Figure 2:**
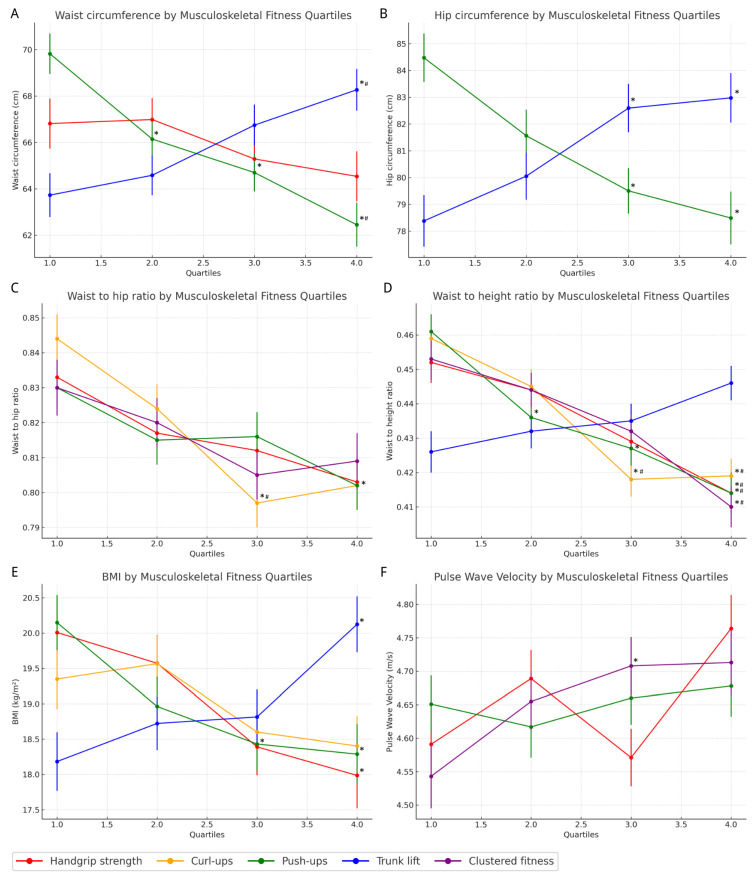
ANCOVA quartiles of muscle strength (HGS, curl-ups, push-ups, trunk lifts and clustered fitness) and cardiovascular risk factors (WC, HC, WHR, WHtR, BMI, PWV). The selection of the exposure variables for cardiovascular risk factors was based on making a significant independent contribution in the linear regression model, as outlined in Table 2. * denotes a significant difference compared to quartile 1. # denotes a significant difference compared to quartile 2.

Figure 2 illustrates the associations between quartiles of MF components and CVD risk factors, as assessed by ANCOVA.

HC was significantly different for trunk lift (*p* < 0.001) and push-up (*p* < 0.001) quartile groups: HC was significantly higher in trunk lift quartile 3 (Q3) and Q4 compared to Q1 (+4.2 to +4.6 cm) and significantly lower in push-up Q3 and Q4 versus Q1 (−5.0 to −6.0 cm) (Figure 2A). Significantly higher WCs were observed across push-ups (*p* < 0.001) and were significantly lower in trunk lifts (*p* < 0.001), with no significant difference for HGS (*p* = 0.129) (Figure 2B). The largest difference was observed between Q4 and Q1 for trunk lifts (+4.5 cm) and push-ups (−7.4 cm). WHR was significantly lower across quartiles of HGS (*p* = 0.027), curl-ups (*p* < 0.001), and push-ups (*p* = 0.015), with no significant difference across quartiles of clustered MF (*p* = 0.058) (Figure 2C). Compared to Q1, curl-ups in Q3 and Q4 were significantly lower (−0.047 to −0.042). WHtR was also significantly lower across HGS, curl-ups, push-ups, and clustered MF (all *p* < 0.001) but higher across trunk lift quartiles (*p* = 0.015) (Figure 2D). Representative contrasts included HGS Q4 vs. Q1 (−0.038), push-up Q4 vs. Q1 (−0.047) and curl-up Q3–Q4 vs. Q1 (−0.042 to −0.040). BMI significantly differed across HGS (*p* = 0.003), push-ups (*p* = 0.002) and trunk lift (*p* < 0.001), but not curl-ups (*p* = 0.061) (Figure 2E). Compared to Q1, BMI was significantly lower in HGS Q4 (−2.0 kg·m^−2^) and push-up Q3–Q4 (−1.7 to −1.9 kg·m^−2^) and higher in trunk lift Q4 vs. Q1, with +1.9 kg·m^−2^.

PWV was significantly different for the clustered fitness quartile group (*p* = 0.024) and significantly higher in Q3 to Q1 (0.165 m·s^−1^). There were no significant effects for PWV across HGS (*p* = 0.124) and push-up (*p* = 0.547) quartile groups.

## 4. Discussion

This study examined the association between MF and CVD risk factors in adolescents with ConHD. It was found that several MF components demonstrated significant inverse associations with anthropometric indices, including WC, HC, WHR, WHtR and BMI. Specifically, (1) push-ups showed the most consistent pattern across all anthropometric indices; (2) HGS showed significant inverse associations with WC, WHR, WHtR and BMI but no significant association with HC; (3) curl-ups were significantly and inversely related to WHR and BMI; (4) trunk lifts consistently demonstrated a positive and significant relationship with WC, HC, WHtR and BMI; and (5) clustered MF was inversely associated with WHR and WHtR. In contrast, MF variables were not associated with peripheral or central BP, while HGS, push-ups, and clustered fitness showed significant positive associations with PWV. Given that obesity is a central determinant of CVD risks and is modifiable through physical activity interventions that promote fitness, our results highlight the potential value of MF as a practical screening marker for obesity-related risk in adolescents with ConHD. However, the observed positive associations between selected MF variables (HGS, push-ups, and clustered fitness) and PWV indicate that the relationship between MF and arterial stiffness may be more complex in this population. Further investigation is therefore warranted to clarify whether these findings reflect underlying anatomical or developmental characteristics specific to ConHD.

HGS demonstrated significant inverse associations with several anthropometric indices, including WC, WHR, WHtR, and BMI, while showing no significant association with HC, which indicates that HGS primarily reflects central obesity and overall body composition rather than peripheral obesity. This observation is consistent with previous cross-sectional observations in the healthy population [20,21]. In adolescents with ConHD, restricted physical activity and metabolic irregularities predispose them to increased visceral fat accumulation [22]. Although the present study cannot establish causality, enhanced HGS may suppress central obesity by increasing basal metabolic rate and energy expenditure, improving glucose and lipid metabolism, and promoting visceral fat oxidation [23]. In addition, given the high prevalence of central obesity (24.6%) among adolescents with ConHD [24] and its strong association with all-cause mortality [25], HGS may serve as a practical marker for identifying adolescents with ConHD who are at an increased risk of obesity-related CVD.

Beyond HGS, several other MF exposures demonstrated significant inverse associations with multiple anthropometric indices in adolescents with ConHD, albeit with varying patterns. Push-ups emerged as a consistent MF indicator, showing negative associations with HC, WC, WHR, WHtR, and BMI in both regression and quartile analyses. This result indicates that push-ups may be more sensitive than other MF tests in reflecting the effects of obesity on adolescents. A plausible explanation is that push-ups, as a weight-bearing, multi-joint strength endurance exercise, require the coordinated activation of multiple muscle groups and impose greater metabolic and mechanical demands when body fat mass is higher, leading to poorer performance among individuals with higher adiposity. In adolescents with ConHD, this negative association appears to be further amplified by their reduced muscular fitness compared with healthy adolescents, which constrains exercise tolerance and functional capacity. Interestingly, trunk lifts were positively associated with HC, WC, WHtR, and BMI. This is inconsistent with previous research findings that children with a low BMI perform relatively better in trunk extension, while children with a high BMI have an advantage in upper body strength [26]. This may be related to insufficient baseline muscular endurance and cardiovascular limitations. Collectively, these findings underscore the importance of improving MF and systematically incorporating strength training into rehabilitation that may help these adolescents improve MF, thereby alleviating the adverse effects of obesity. However, substantial differences in physical reserves and exercise tolerance across ConHD types, surgical histories, and functional classifications may lead to heterogeneity in the association between MF and obesity, which could not be examined in the present study due to the limited sample size. Therefore, future multi-centre studies with larger samples are warranted to determine whether the MF–obesity relationship varies across disease subtypes and functional strata.

Allometric HGS, push-ups and clustered MF were positively associated with PWV in the current study. This finding stands in marked contrast to observations in healthy populations. A review demonstrated that MF is inversely correlated with arterial stiffness, as measured by PWV in healthy individuals [27]; the cross-sectional studies included in this review demonstrating this negative relationship were conducted primarily in middle-aged and older populations, in whom sarcopenia and age-related vascular degeneration may underlie the association. In contrast, our cohort comprised adolescents with congenital heart disease, characterised by altered haemodynamics and early vascular remodelling rather than degenerative arterial change. Furthermore, previous studies adjusted for BMI, whereas BMI was not included as a covariate in the present analysis due to its role as an outcome variable. These differences in population characteristics and model specification may partly explain the divergent findings. Despite adjusting HGS for body size to minimise anthropometric coupling, this expected inverse pattern was not observed. Compared to healthy adolescents, adolescents with ConHD show increased arterial stiffness already in childhood [28]. PWV is primarily influenced by disease-specific structural and surgical factors rather than behavioural or fitness-related determinants [29]. Increased arterial stiffness in this population has been attributed to abnormalities in smooth muscle morphology, elastin and collagen organisation, surgical scarring, fibrotic tissue formation, and the presence of grafts or patch materials, all of which compromise the natural buffering capacity of the arterial wall [30]. Consequently, higher MF in this context may simply reflect greater body size or more advanced developmental trajectories or underlying anatomical and developmental characteristics specific to ConHD, rather than any detrimental vascular consequence of higher MF. It was for this reason that HGS was allometrically scaled; this study is the first to report this variable to better account for body size differences. Nevertheless, longer-term longitudinal follow-up is required to clarify the temporal direction and clinical significance of the MF and PWV association in this population.

No significant associations were observed between MF and peripheral or central blood pressure. This result is consistent with previous findings in healthy paediatric populations, in which the relationship between MF and blood pressure has been weak or inconsistent [31]. Throughout childhood and adolescence, SBP and DBP rise largely as a function of increases in height, body mass, and cardiac output, reflecting the rapid growth of the cardiovascular system. Moreover, the limited variability (peripheral SBP 111 [103–117] mmHg; peripheral DBP 63 [59–69] mmHg) in blood pressure within our cohort may have reduced the sensitivity to detect MF-related differences. In addition, blood pressure regulation in this population is often influenced by structural and haemodynamic factors inherent to congenital heart defects and their surgical repairs, such as reduced aortic compliance or residual valve dysfunction, that may override peripheral vascular adaptations associated with MF [32]. These factors showed that BP in adolescents with ConHD is predominantly shaped by growth-related and disease-specific cardiovascular determinants, leaving little scope for MF to exert a measurable independent influence on either peripheral or central blood pressure.

A key strength of this study is the comprehensive assessment of MF using multiple complementary field-based tests, including HGS, push-ups, curl-ups, and trunk lifts, which allowed a broader and more meaningful characterisation of neuromuscular function than reliance on a single indicator. As with all studies, several limitations should be acknowledged. The cross-sectional design precludes causal inferences. In addition, although 645 participants were originally recruited, only 355 were included in the final analyses due to missing data. Although independent-sample *t*-tests comparing MS between included and excluded participants revealed no statistically significant differences, the possibility of selection bias cannot be entirely ruled out. The available sample size also limited the ability to conduct stratified analyses across specific ConHD subtypes, surgical histories, or functional classes, leaving potential heterogeneity in MF and obesity relationships unexamined. However, we controlled for these factors without our analyses. Finally, the performance-based MF tests used in this study may be influenced by participant motivation, coordination, and familiarity with the testing procedures, particularly in younger or more clinically complex adolescents.

## 5. Conclusions

In adolescents with ConHD, the consistent associations between MF and anthropometric indicators, especially HGS and push-ups, reflect the predictability of MF on obesity-related CVD risk. MF showed no significant associations with blood pressure but positive associations with PWV; this finding should not be interpreted as evidence of detrimental vascular effects. Rather, it may reflect physiological growth processes, body size influences, or ConHD-specific haemodynamic adaptations. Collectively, these findings highlight the dual role of MF in ConHD: as a sensitive indicator of obesity-related CVD risk and as a complex correlate of cardiovascular health. From a clinical perspective, systematically increasing MF in rehabilitation may provide a feasible strategy to mitigate obesity-related CVD risks in this population. Future longitudinal studies are warranted to confirm that these associations are stable and evaluate the effectiveness of strength training tailored to adolescents with ConHD.

## Figures and Tables

**Table 1:** Descriptive characteristics of participants.

Variables	Total *N* = 355	Simple 91 (25.6%)	Moderate 109 (30.7%)	Complex 155 (43.7%)
**Sex, *n* (%)**				
Male	201 (56.6%)	43 (21.4%)	61 (30.3%)	97 (48.3%)
Female	154 (43.4%)	48 (31.2%)	48 (31.2%)	58 (37.7%)
Age (years)	12.4 [9.3–15.3]	13.3 [9.1–15.4]	12.1 [8.9–14.7]	12.5 [9.8–15.8]
**ConHD Category, *n* (%)**				
Aortic Isthmus Stenosis	35 (9.9%)	1	28	6
Aortic Stenosis	46 (13.0%)	23	16	7
ASD	40 (11.3%)	39	1	0
AVSD	11 (3.1%)	0	10	1
CCTGA	5 (1.4%)	0	0	5
EBS	6 (1.7%)	0	6	0
PFO	4 (1.1%)	4	0	0
Pulmonary Stenosis	17 (4.8%)	2	11	4
Pulmonary Vein Anomaly	6 (1.7%)	1	4	1
TAC	9 (2.5%)	0	0	9
TGA n. Rastelli	6 (1.7%)	0	0	6
TGA n. Switch	35 (9.9%)	0	0	35
ToF	51 (14.4%)	0	26	25
UVH	52 (14.6%)	0	0	52
VSD	29 (8.2%)	21	7	1
Zyanotisch	3 (0.8%)	0	0	3
**Medication, *n* (%)**				
None	259 (73.0%)	83	91	85
Anticoagulants	46 (12.9%)	1	4	41
ACE Inhibitors/ARBs	20 (5.7%)	2	3	15
Beta-blockers	17 (4.7%)	1	3	13
Antiplatelet Agents	9 (2.6%)	0	2	7
Diuretics	17 (4.8%)	1	3	13
**Number of Heart Surgeries, *n* (%)**				
0	102 (28.7%)	71	31	0
1	129 (36.3%)	17	63	49
2	50 (14.1%)	2	11	37
3	54 (15.2%)	0	2	52
4	11 (3.1%)	0	2	9
5	7 (2.0%)	0	0	7
6	1 (0.3%)	0	0	1
N/A	1 (0.3%)	1	0	0
**Number of Catheters, *n* (%)**				
0	219 (61.7%)	67	71	81
1	96 (27.0%)	22	25	49
2	20 (5.6%)	1	9	10
3	13 (3.7%)	0	3	10
4	4 (1.1%)	0	0	4
6	1 (0.3%)	0	0	1
7	1 (0.3%)	0	1	0
N/A	1 (0.3%)	1	0	0
**Handiness, *n* (%)**				
Right	300 (84.5%)	78	93	129
Left	43 (12.1%)	11	11	21
N/A	12 (3.4%)	2	5	5
Body Mass (kg)	44.0 [28.5–60.0]	43.0 [28.7–60.0]	41.0 [27.1–59.0]	44.0 [28.5–60.0]
Height (cm)	153.5 [133.0–167.0]	159.0 [134.0–169.0]	151.0 [133.0–164.0]	153.5 [133.0–168.0]
**Musculoskeletal Fitness**				
Handgrip Strength Right mean (kg)	18.3 [11.0–28.3]	22.0 [11.7–31.0]	16.7 [10.3–26.5]	18.0 [11.0–29.0]
Handgrip Strength Left Mean (kg)	17.3 [10.7–26.7]	21.3 [11.3–29.3]	16.0 [9.8–22.8]	17.0 [10.3–26.7]
Handgrip Strength Mean (kg)	18.1 [10.9–27.5]	22.4 [11.9–29.9]	16.5 [10.1–24.3]	18.0 [10.7–28.5]
Curl-Ups (n)	13 [4–28]	16 [5–35]	13 [4–24]	11 [3–26]
Push-Ups (n)	7 [1–15]	10 [3–15]	6 [1–15]	7 [0–15]
Trunk Lift (cm)	22.0 [18.0–27.0]	22.0 [18.0–27.0]	21.5 [18.0–28.0]	21.0 [17.0–24.0]
**Cardiovascular Risk Factors**				
**Weight status, *n* (%)**				
Normal Weight	294 (82.8%)	76	90	128
Overweight	44 (12.4%)	16	16	12
Obesity	17 (4.8%)	5	6	6
Waist Circumference (cm)	65.0 [56.5–72.0]	66.0 [56.0–70.0]	62.5 [55.5–71.0]	65 [57.0–73.0]
Hip Circumference (cm)	81.0 [69.0–91.5]	86.0 [70.5–92.0]	79.0 [67.8–91.3]	81.0 [68.0–91.0]
Waist-to-Hip Ratio	0.81 [0.77–0.85]	0.79 [0.75–0.84]	0.80 [0.76–0.85]	0.82 [0.78–0.86]
Waist-to-Height Ratio	0.43 [0.40–0.46]	0.42 [0.39–0.44]	0.43 [0.40–0.46]	0.43 [0.40–0.47]
BMI (kg/m^2^)	18.37 [15.86–20.90]	19.27 [16.38–21.01]	17.59 [15.76–21.11]	18.17 [15.61–20.62]
Peripheral SBP (mmHg)	111 [103–117]	110 ± 11	111 ± 11	112 ± 10
Peripheral DBP (mmHg)	63 [59–69]	63 [60–68]	65 ± 8	64 ± 8
Central SBP (mmHg)	100 [93–108]	101 ± 11	101 ± 11	101 ± 12
Central DBP (mmHg)	65 [60–70]	65 ± 8	66 ± 8	65 ± 8
Heart rate (bpm)	72 ± 12	72 ± 12	74 ± 11	71 ± 13
Pulse Wave Velocity (m/s)	4.60 [4.31–4.92]	4.57 ± 0.46	4.62 ± 0.43	4.69 ± 0.48
Carotid Intima Media Thickness (mm)	0.450 [0.430–0.480]	0.448 [0.430–0.475]	0.448 [0.429–0.471]	0.454 [0.427–0.479]

ConHD, congenital heart disease; AVSD, atrioventricular septal defect; CCTGA, congenitally corrected transposition of the great arteries; EBS, Ebstein’s anomaly; PFO, patent foramen ovale; TAC, truncus arteriosus communis; TGA n. Rastelli, transposition of the great arteries repaired with the Rastelli procedure; TGA n. Switch, transposition of the great arteries repaired with the arterial switch operation; ToF, tetralogy of Fallot; UVH, univentricular heart; VSD, ventricular septal defect; BMI, body mass index; Peripheral SBP, peripheral systolic blood pressure; Peripheral DBP, peripheral diastolic blood pressure; central SBP, central systolic blood pressure; central DBP, central diastolic blood pressure; ACE inhibitors, angiotensin-converting enzyme inhibitors; ARBs, angiotensin receptor blockers.

**Table 2:** The linear regression between musculoskeletal fitness and cardiovascular risk factors.

	Handgrip Strength (Allometric)	Curl-Ups (Max Repetition)	Push-Ups (Max Repetition)	Trunk Lift (cm)	Clustered Fitness
Peripheral SBP (mmHg)	1.794 (−2.374–5.961) *p* = 0.398	0.011 (0.039–0.145) *p* = 0.712	0.019 (−0.088–0.126) *p* = 0.724	0.102 (−0.158–0.262) *p* = 0.211	0.290 (−0.203–0.782) *p* = 0.248
Peripheral DBP (mmHg)	−0.712 (−3.993–2.569) *p* = 0.67	0.017 (−0.027–0.061) *p* = 0.453	−0.065 (−0.148–0.019) *p* = 0.131	0.072 (−0.054–0.198) *p* = 0.261	−0.001 (−0.389–0.388) *p* = 0.997
Central SBP (mmHg)	1.827 (−2.39–6.043) *p* = 0.395	0.009 (−0.048–0.066) *p* = 0.758	0.058 (−0.05–0.166) *p* = 0.293	0.154 (−0.007–0.316) *p* = 0.061	0.434 (−0.063–0.931) *p* = 0.087
Central DBP (mmHg)	−0.599 (−3.983–2.875) *p* = 0.728	0.017 (−0.079–0.063) *p* = 0.47	−0.07 (−0.156–0.017) *p* = 0.114	0.076 (−0.054–0.206) *p* = 0.252	−0.002 (−0.402–0.399) *p* = 0.993
Hip Circumference (cm)	−1.133 (−4.782–2.516) *p* = 0.542	0.003 (−0.046–0.053) *p* = 0.9	**−** **0.167** **(** **−** **0.259–** **−** **0.075)** ***p*** = 0.001 *	**0.261** **(0.123–0.399)** ***p*** = 0.001 *	−0.021 (−0.454–0.411) *p* = 0.923
Waist Circumference (cm)	**−** **4.467** **(** **−** **8.006–** **−** **0.928)** ***p*** = 0.014 *	−0.039 (−0.089–0.009) *p* = 0.109	**−** **0.205** **(** **−** **0.294–** **−** **0.116)** ***p*** = 0.001 *	**0.255** **(0.12–0.39)** ***p*** = 0.001 *	−0.402 (−0.823–0.019) *p* = 0.061
Waist-to-Hip Ratio	**−** **0.039** **(** **−** **0.066–** **−** **0.012)** ***p*** = 0.005 *	**0** **(** **−** **0.001–0)** ***p*** = 0.01 *	**−** **0.001** **(** **−** **0.001–0)** ***p*** = 0.032 *	0 (−0.001–0.002) *p* = 0.385	**−** **0.004** **(** **−** **0.008–** **−** **0.001)** ***p*** = 0.008 *
Waist-to-Height Ratio	**−** **0.052** **(** **−** **0.073–** **−** **0.032)** ***p*** = 0.001 *	**−** **0.001** **(** **−** **0.001–0)** ***p*** = 0.001 *	**−** **0.001** **(** **−** **0.002–** **−** **0.001)** ***p*** = 0.001 *	**0.001** **(0–0.002)** ***p*** = 0.011 *	**−** **0.006** **(** **−** **0.008–** **−** **0.003)** ***p*** = 0.001 *
BMI (kg·m^−2^)	**−** **3.115** **(** **−** **4.642–** **−** **1.588)** ***p*** = 0.001 *	**−** **0.024** **(** **−** **0.045–** **−** **0.003)** ***p*** = 0.025 *	**−** **0.05** **(** **−** **0.09–** **−** **0.01)** ***p*** = 0.014 *	**0.119** **(0.06–0.178)** ***p*** = 0.001 *	−0.154 (−0.338–0.030) *p* = 0.101
Heart Rate (bpm)	0.396 (−4.724–5.516) *p* = 0.879	−0.049 (−0.118–0.02) *p* = 0.164	−0.034 (−0.165–0.098) *p* = 0.616	0.085 (−0.112–0.282) *p* = 0.395	−0.115 (−0 721–0.491) *p* = 0.710
Pulse Wave Velocity (m/s)	**0.18** **(0.014–0.346)** ***p*** = 0.033 *	0.001 (−0.002–0.003) *p* = 0.53	**0.004** **(0–0.009)** ***p*** = 0.041 *	0.006 (0–0.013) *p* = 0.054	**0.028** **(0.008–0.047)** ***p*** = 0.006 *
Carotid Intima Media Thickness (mm)	0.009 (−0.008–0.027) *p* = 0.291	0.0001 (−0.0001–0.0004) *p* = 0.324	0.0004 (−0.0005–0.001) *p* = 0.081	0.00007 (−0.001–0.001) *p* = 0.837	0.002 (0–0.004) *p* = 0.104

Data are reported as the standardised regression coefficient point estimate with the associated *p* value. * And bold numbers are used to highlight a significant result. Peripheral SBP, peripheral systolic blood pressure; Peripheral DBP, peripheral diastolic blood pressure; Central SBP, central systolic blood pressure; Central DBP, central diastolic blood pressure; BMI, body mass index.

## Data Availability

No data are available. For the purpose of open access, the author has applied a Creative Commons Attribution (CC BY) licence to any Author Accepted Manuscript version arising from this submission.

## Supplementary Materials

The following supporting information can be downloaded at https://www.mdpi.com/article/10.3390/jcm15082863/s1, Table S1: STROBE checklist; Table S2: Detailed specification of musculoskeletal fitness tasks; Table S3: The *t*-test results between the final analysis participants and the excluded participants without cardiovascular risk factors. References [14,33] is cited in Supplementary Materials.

## Abbreviations

The following abbreviations are used in this manuscript:

ConHD	Congenital heart disease
CVD	Cardiovascular disease
MVPA	Moderate-to-vigorous physical activity
MF	Musculoskeletal fitness
PA	Physical activity
WC	Waist circumference
BP	Blood pressure
PWV	Pulse wave velocity
HP	Hip circumference
WHR	Waist-to-hip ratio
WHtR	Waist-to-height ratio
BMI	Body mass index
SBP	Systolic blood pressure
DBP	Diastolic blood pressure
HR	Heart rate
cIMT	Carotid intima media thickness
HGS	Handgrip strength
ANCOVA	Analysis of covariance
