# Supplementary material for: Predictive Value of Musculoskeletal Fitness for Cardiovascular Risk Factors in Adolescents with Congenital Heart Disease: A Cross-Sectional Study
Source: J Clin Med. 2026 Apr 9;15(8):2863. doi: 10.3390/jcm15082863 (PMC13116863; doi:10.3390/jcm15082863)
Supplement: Supplementary file 1 [file jcm-15-02863-s001.zip › Kunyu association study manuscript supplement table S2.pdf]

Supplement Table S2: Detailed specification of musculoskeletal fitness tasks

Test	Skill	Task	Assessment	Impression
**Curl-Up**	Abdominal strength and muscular endurance	Lying in supine position with flexed knees at an angle of around 140° and feet placed on the mat. The arms are stretched out on the level of the knees, while the upper body moves slowly towards the knees and afterwards back to the mat	The exercise should be performed until exhaustion, a maximum of 75 repetitions or until the second formal correction is made. Formal corrections are necessary in case of feet leaving the mat or if pauses are made	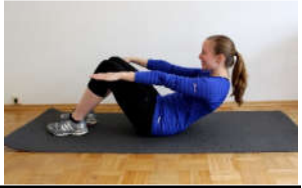
**Push-Up**	Upper body strength and muscular endurance	First adopting a prone position, with arms straight and under the shoulders. Back and legs have to be also straight. Then pushing up and down with arms till elbows are flexed to an angle of 90° until exhaustion.	The exercise should be performed until exhaustion or until a correct performance is no longer possible. Formal corrections are allowed if extending arms fully straightened or flexing arms not achieving a 90° angle in the elbows. The score of valid repetition is the number recorded for push-ups.	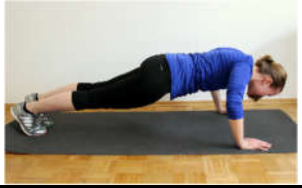
**Trunk Lift**	Trunk extensor strength and flexibility	Lying in prone position with toes pointed and hands under the thighs, then lifting upper body to maximum of 12 inches (30.5 cm) without bouncing movements while looking towards the floor	The patients should have their head extended from the spine and look straight to the mat, while lifting the upper body slowly. Distance between the patient's chin and the mat is assessed during pause in the the lifted position.The patient has two trials and the better one is recorded	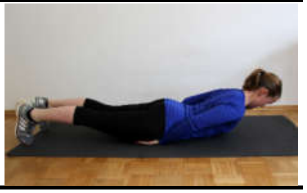
