# Supplementary material for: Predictive Value of Musculoskeletal Fitness for Cardiovascular Risk Factors in Adolescents with Congenital Heart Disease: A Cross-Sectional Study
Source: J Clin Med. 2026 Apr 9;15(8):2863. doi: 10.3390/jcm15082863 (PMC13116863; doi:10.3390/jcm15082863)
Supplement: Supplementary file 1 [file jcm-15-02863-s001.zip › Kunyu association study manuscript supplement table S3.pdf]

Supplement Table S3: The T-test results between the Final analysis participants and the Excluded participants without cardiovascular risk factors.

Variables	Final analysis participants (N=355)		Excluded participants without cardiovascular risk factors (N=260)		P-value	Effect size
	mean	SD	mean	SD		
**Sex, (female/male), n/n**	154/201		99/161		0.187	N/A
**Age (years)**	12.5	3.5	12.3	3.5	0.583	0.05
**Weight (kg)**	45.73	18.82	43.45	18.14	0.132	0.12
**Height (cm)**	151.6	19.5	149.7	19.3	0.236	0.10
**Muscle strength**						
**Handgrip strength mean (kg)**	20.31	11.28	19.31	10.97	0.270	0.09
**Curl-Ups (n)**	19	21	18	20	0.385	0.07
**Push-Ups (n)**	10	11	10	9	0.774	0.02
**Trunk Lift (cm)**	22.7	6.8	21.4	7.1	**0.029***	0.18
**Sit and reach (cm)**	-1.5	8.7	-2.8	9.4	0.079	0.14
**Shoulder stretch (cm)**	-13.2	7.3	-13.8	7.9	0.284	0.09

Supplementary Table. Comparison of anthropometrics and musculoskeletal fitness characteristics between participants included in the final analysis and those excluded due to missing cardiovascular risk factor data. Values are presented as mean ± SD unless otherwise stated. P-values are derived from independent-samples t-tests (or chi-square test for sex).

Effect size was calculated as Cohen's d for continuous variables:

$$Cohen's\ d = \frac{Mean_{final} - Mean_{exclud}}{SD_{pooled}}$$
